# The Keap1/Nrf2 Signaling Pathway in the Thyroid—2020 Update

**DOI:** 10.3390/antiox9111082

**Published:** 2020-11-04

**Authors:** Christina Thanas, Panos G. Ziros, Dionysios V. Chartoumpekis, Cédric O. Renaud, Gerasimos P. Sykiotis

**Affiliations:** 1 Service of Endocrinology, Diabetology and Metabolism, Lausanne University Hospital and University of Lausanne, CH-1011 Lausanne, Switzerland; christinethanas@gmail.com (C.T.); panos.ziros@chuv.ch (P.G.Z.); dchart@upatras.gr (D.V.C.); cedric.renaud@unil.ch (C.O.R.); 2 Division of Endocrinology, Department of Internal Medicine, School of Medicine, University of Patras, GR-26500 Patras, Greece

**Keywords:** thyroid, Nrf2 (nuclear factor erythroid 2-related factor 2), Keap1 (Kelch-like ECH-associated protein 1), thyroglobulin, oxidative stress, goiter, antioxidant

## Abstract

The thyroid gland has a special relationship with oxidative stress. On the one hand, like all other tissues, it must defend itself against reactive oxygen species (ROS). On the other hand, unlike most other tissues, it must also produce reactive oxygen species in order to synthesize its hormones that contribute to the homeostasis of other tissues. The thyroid must therefore also rely on antioxidant defense systems to maintain its own homeostasis in the face of continuous self-exposure to ROS. One of the main endogenous antioxidant systems is the pathway centered on the transcription factor Nuclear factor erythroid 2-related factor 2 (Nrf2) and its cytoplasmic inhibitor Kelch-like ECH-associated protein 1 (Keap1). Over the last few years, multiple links have emerged between the Keap1/Nrf2 pathway and thyroid physiology, as well as various thyroid pathologies, including autoimmunity, goiter, hypothyroidism, hyperthyroidism, and cancer. In the present mini-review, we summarize recent studies shedding new light into the roles of Keap1/Nrf2 signaling in the thyroid.

## 1. Introduction and Overview

When the cellular amounts of reactive oxygen species (ROS) are retained within physiological levels, ROS can function as signaling molecules that play key roles in cellular processes. In contrast, when the amounts of ROS exceed physiological levels, they are considered potent DNA-, protein-, and lipid-damaging agents that cells need to defend themselves against. Oxidative stress (OS) is the imbalance that occurs when the concentration of ROS overcomes the cell’s ability to detoxify them using its endogenous antioxidant systems [1]. OS can initiate and maintain cellular damage, including genomic instability, and it has been incriminated as a causative or exacerbating factor in a variety of pathological conditions. Disorders linked to OS can affect many tissues, especially those that are in direct contact with the external environment (e.g., lung), generate high amounts of free radicals as either mediators or side-products of their normal physiology (e.g., muscle), have roles in detoxification (e.g., liver), or are, in general, especially sensitive to free radicals (e.g., neurons) [2].

**Figure 1:**
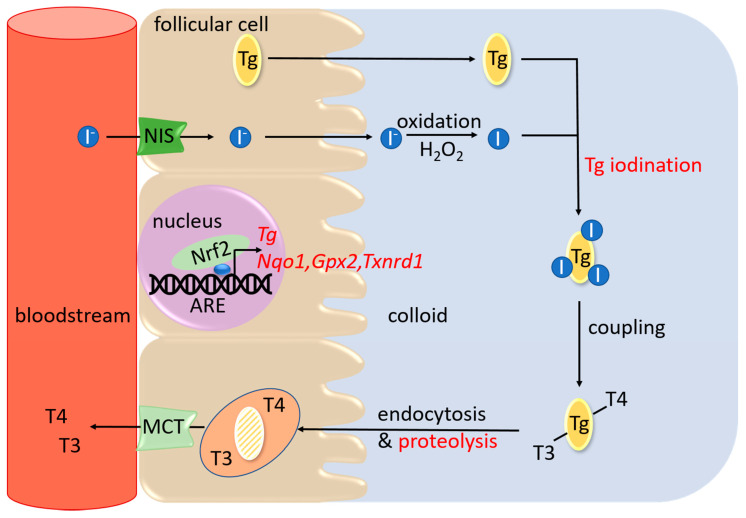
Schematic simplified representation of thyroid hormone synthesis. Iodide (I^−^) enters thyroid follicular cells by active transport via the sodium (Na^+^)-iodide symporter (NIS), and is then exported via other channels into the follicular lumen (the colloid area), where it is oxidized to iodine (I) and used in the iodination of thyroglobulin (Tg). Coupling between di- and/or mono-iodinated tyrosines of Tg produces the thyroid hormones T4 and T3, attached to the Tg backbone. Tg is then endocytosed into the thyroid follicular cells, where it is degraded inside lysosomes, ultimately releasing T4 and T3 into the blood circulation via a monocarboxylate transporter (MCT). Steps affected by Keap1/Nrf2 signaling are highlighted in red font. They include: (i) a positive effect on the transcription of the gene encoding Tg; (ii) a positive effect on the transcription of antioxidant and selenoprotein genes such as *Nqo1* (NAD(P)H quinone dehydrogenase 1), *Gpx2* (glutathione peroxidase 2), and *Txnrd1* (thioredoxin reductase 1); (iii) a negative effect on Tg iodination; (iv) a positive effect on Tg proteolysis.

Hydrogen peroxide (H_2_O_2_) is a relatively stable ROS that is important for many cellular functions, including signaling, host defense, wound healing, cell death, etc. [3]. Physiological levels of H_2_O_2_ are especially important for the normal functioning and hormone-producing capacity of the thyroid gland. The thyroid is an endocrine gland responsible primarily for the production of the thyroid hormones thyroxine (T4) and triiodothyronine (T3), which act on all cells in the body to regulate development, growth, and metabolism. Synthesis of these hormones takes place in special spherical structures called thyroid follicles, whose periphery is lined by thyroid follicular cells. Thyroid hormone synthesis is a complex and multistep process that comprises a series of redox reactions that use H_2_O_2_ as the primary oxidative agent (Figure 1) [4]. Considering that the levels of H_2_O_2_, similar to those of other ROS, should remain balanced in order to prevent OS, thyroid follicular cells also need to have a potent antioxidant system, including a cellular sensor for oxidants and a mediator of the antioxidant response. In our studies over the last few years, we have shown that the antioxidant signaling pathway comprising the transcription factor Nrf2 (Nuclear factor erythroid 2-related factor 2) and its cytoplasmic inhibitor Keap1 (Kelch-like ECH-associated protein 1) is a main system employed by the thyroid for its antioxidant defense [5], similar to other tissues [6].

Under normal conditions, Keap1 acts as an adaptor that targets Nrf2 for poly-ubiquitination by Cullin 3-based ubiquitin E3 ligase (Cul3) and subsequent degradation by the proteasome. The thiol groups on specific Keap1 cysteines react readily with oxidants, including H_2_O_2_, and their oxidation impairs the presentation of Nrf2 to Cul3 for poly-ubiquitination [7,8]. Nrf2 can then accumulate in the nucleus, where it plays its role as a transcription factor by binding to DNA sequences called Antioxidant Response Elements (AREs) as a dimer with members of the small musculoaponeurotic fibrosarcoma (Maf) family of proteins [9,10]. In this manner, activated Nrf2 induces the transcription of a large battery of genes encoding antioxidant enzymes and various other cell-protective molecules [6].

Animal models have firmly established the importance of Keap1/Nrf2 signaling as an antioxidant response system. Nrf2 was found to be dispensable for mouse development and growth, meaning that Nrf2 knockout (KO) adult mice are viable and fertile [11]. On the other hand, Keap1 KO mice die shortly after birth [12], precluding their use for physiological studies; however mice expressing lower levels of Keap1, referred to as Keap1 hypomorphic or Keap1 knockdown (Keap1^KD^) mice, are viable and fertile [13]. Research using the aforementioned mouse models, along with respective cellular Nrf2 gain- and loss-of-function models, has elucidated many roles of Keap1/Nrf2 signaling in pathophysiology, and has highlighted the Keap1/Nrf2 pathway as a plausible therapeutic target for a wide spectrum of OS-related diseases affecting various tissues [14]. As mentioned, the thyroid, and specifically the follicular cells which continuously produce and are exposed to H_2_O_2_, need to be able to adapt and protect themselves against OS. However, information about the Keap1/Nrf2 pathway in the physiology and pathophysiology of the thyroid has emerged only in the last few years, as summarized in our previous review [5]. As new knowledge in this area has become available over the last two years, we provide here an update on the topic of Keap1/Nrf2 signaling in the thyroid, focusing on the latest studies by our group and others.

As mentioned, normal production of T4 and T3 by the thyroid is essential for homeostasis because they act throughout the body to control development, growth, and metabolism [15,16,17]. As all tissues depend on thyroid hormones for normal functioning, thyroid dysfunction with hormonal over- or underproduction can be a cause of multiple symptoms and clinical signs. Thyroid diseases are actually common in the population, and the most frequent ones are (i) hypothyroidism, which refers to hypofunction of the gland, with thyroid hormone underproduction [18]; (ii) thyrotoxicosis, which refers to increased levels of thyroid hormones due to either true overproduction (hyperthyroidism) or increased liberation due to inflammation and destruction of the follicles [19,20]; (iii) autoimmune thyroid disease, which is most often associated with hypothyroidism (Hashimoto’s thyroiditis, HT) but in other cases with hyperthyroidism (Graves’ disease, GD) [21]; (iv) thyroid enlargement (also termed goiter), either in a diffuse form, as is the case in GD and occasionally also in HT, or in the form of single or multiple thyroid nodules (tumors); the latter are usually benign, even though thyroid carcinomas are not very rare either [22,23]. Recent studies have highlighted links between the Keap1/Nrf2 pathway and thyroid physiology as well as various of the aforementioned pathologies; these links will be briefly reviewed in the respective following sections.

## 2. Thyroid Physiology

Our group has shown that, similar to its function in other tissues, Nrf2 is a key antioxidant player in the thyroid. In studies using Nrf2 KO mice, mice with tissue-restricted deletion of Nrf2 (using Pax8-Cre recombinase that is expressed primarily in the thyroid and the kidney), and thyroid follicular cell lines with inactivated Nrf2 or Keap1 (using the CRISPR/Cas9 method), we showed that Nrf2 activity correlated positively with the mRNA expression levels of antioxidant and/or selenoprotein genes such as *Nqo1* (NAD(P)H quinone dehydrogenase 1), *Gpx2* (glutathione peroxidase 2), and *Txnrd1* (thioredoxin reductase 1), as well as with the protein levels of Nqo1 and selenoprotein S (SelS) [2]. More recently, we confirmed this further by demonstrating increased mRNA expression levels of *Nqo1*, *Gpx2* and *Txnrd1* as well as increased protein abundance of Nqo1 in the thyroids of Keap1^KD^ mice [24]. We also showed that the antioxidant function of Nrf2 is triggered by exposure to supraphysiological amounts of iodide, in order to protect thyroid follicular cells from OS [2]. Another group has also shown that iodide excess activates Nrf2 in the thyroid of rats [25].

In addition to its antioxidant function in thyroid follicular cells, Nrf2 was also found to impact two central aspects of thyroid hormone synthesis. On the one hand, it directly upregulates the transcription of the gene encoding thyroglobulin (Tg, the precursor protein of thyroid hormones) via two AREs in its upstream enhancer, and thereby positively regulates the protein abundance of Tg in the thyroid [2]. Interestingly, one of these AREs harbors a rare single-nucleotide polymorphism (SNP) in humans; using reporter gene assays in thyroid follicular cell lines, we showed that the minor allele of this SNP abrogated the basal and inducible transcriptional activity of the enhancer, thus behaving as a complete loss-of-function mutation [26]. Furthermore, by analyzing data from a genome-wide association study, we found statistically significant associations between circulating Tg levels (Tg is also partly secreted in the bloodstream) and common SNPs in the genes encoding Nrf2 (*NFE2L2*) and Keap1 (*KEAP1*), including, notably, a known functional SNP in the promoter of *NFE2L2* [26].

**Figure 2:**
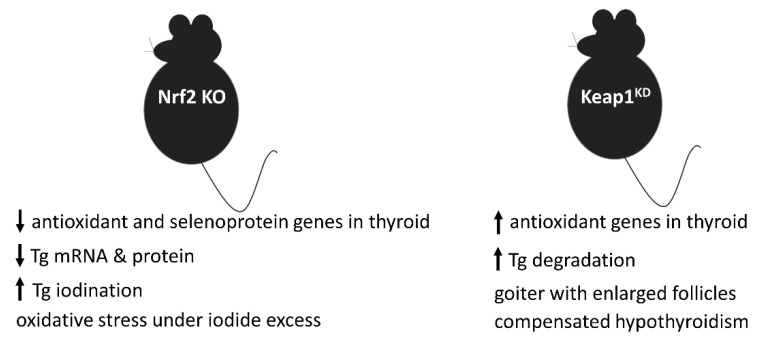
Schematic summary of the molecular, cellular, and/or clinical phenotypes observed in Nrf2 KO mice and in Keap1^KD^ mice (↑, increased; ↓, decreased).

On the other hand, in studies in mice, we found that Nrf2 suppresses the iodination of Tg, both under normal conditions and under excess iodide conditions [2]. Specifically, Nrf2 KO mice showed higher levels of iodinated Tg under normal conditions [2]. Exposure to excess iodide normally suppresses Tg iodination to prevent excess thyroid hormone synthesis that could lead to hyperthyroidism; this phenomenon is part of the mechanisms underlying the so-called autoregulation of the thyroid gland [27]. Notably, in response to iodide overload, Nrf2 KO mice showed even higher levels of iodinated Tg [2], suggesting that Nrf2 has a role in thyroid autoregulation. Although the precise mechanisms underlying this effect have not yet been elucidated, we found that treatment of thyroid follicular cell lines with 2-iodohexadecanal, an iodolipid considered to be one of the main mediators of thyroid autoregulation [28], activated Nrf2, similar to iodide treatment [2]. Recently, we analyzed the transcriptomic response of the mouse thyroid to iodide overload using next-generation RNA sequencing in WT and Nrf2 KO mice. The results confirmed that iodide overload induces the Nrf2 cytoprotective response. The results also showed that iodide upregulates inflammatory, immune, and fibrosis pathways, and that these responses are exacerbated in the absence of Nrf2 [29]. The phenotypes of Nrf2 KO and Keap1^KD^ mice are summarized in Figure 2.

One difference between the thyroid and other tissues is that ROS are not primarily a byproduct of its physiology, but an indispensable part of it. As shown in Figure 1, thyroid follicular cells actively produce H_2_O_2_ to facilitate a cascade of redox reactions that sequentially oxidize iodide, iodinate tyrosine residues within Tg, and couple iodinated tyrosine residues of Tg to each other to form T4 and T3. Interestingly, using cell lines (of non-thyroidal origin) and mouse embryonic fibroblasts expressing a series of Keap1 mutants, it was recently shown that four specific cysteines of Keap1 (Cys226, Cys613, Cys622, and Cys624) are important for sensing H_2_O_2_ [30]. The same study also showed that there exists a fail-safe mechanism in which specific combinations of these cysteines can form a disulfide bond to sense H_2_O_2_. Moreover, this sensing mechanism appears to be distinct from that triggered by other Nrf2 inducers, such as electrophiles [30]. The implications of these recent findings for thyroid physiology are unknown, but the thyroid might serve as an interesting model to address their physiological relevance, since H_2_O_2_ is directly implicated in thyroid hormone synthesis.

## 3. Overt Hypothyroidism and Thyroid Autoimmunity

Hypothyroidism occurs when the amount of thyroid hormones is not sufficient to match the needs of the various tissues. Its clinical manifestations may not always be clinically noticeable at early stages; they range from asymptomatic to life-threatening, and some of the most common ones include fatigue, lethargy, cold intolerance, weight gain, constipation, hoarse voice, and dry skin, but the clinical presentation can be quite variable [18]. One of the common causes of hypothyroidism is HT, an autoimmune disease that leads to lymphocytic infiltration and inflammation of the thyroid as well as thyroid follicular cell apoptosis. Like most other autoimmune diseases, HT is a multifactorial condition influenced by environmental and genetic factors. Among the latter, several SNPs have been found to modulate the risk of developing HT; they tend to reside in genes that are thyroid-specific and/or related to autoimmunity, inflammation, or cellular defense to stress [31].

Like iodine, selenium is another trace element with high concentration in the thyroid, and it is a component of various proteins (selenoproteins) with antioxidant and anti-inflammatory functions in various tissues, including the thyroid; interestingly, selenoprotein genes with homeostatic roles in the thyroid include the Nrf2-regulated genes *Gpx2* and *Txnrd1* [2]. A functional SNP (rs28665122) in the promoter of the gene encoding selenoprotein S (*SELENOS*) has been shown to increase the risk of HT, at least in some ethnic populations [32], though not in others [33]. In the Portuguese population where the *SELENOS* promoter SNP was originally found to increase the risk of HT [32], we recently found that this risk was further modulated by the genotype of the three functional SNPs (rs35652124, rs6706649, and rs6721961) in the promoter of *NFE2L2* [34]. Specifically, increased HT risk was associated with the combined presence of minor alleles of the functional SNPs of the *SELENOS* and *NFE2L2* promoters, whereas the presence of only major alleles of the *NFE2L2* SNP abolished the risk associated with the *SELENOS* SNP minor allele [34]. We further found that, in Nrf2 KO mice, the expression levels of selenoprotein S in thyroid follicular cells were reduced; conversely, in thyroid follicular cell lines, experimentally reducing the expression of selenoprotein S reduced the activity of Nrf2. These findings indicate that the basis for the genetic interaction between *NFE2L2* and *SELENOS* in modulating the risk of HT might be related to a bidirectional positive feedback between the Nrf2 and selenoprotein S pathways [34]. Of note, the HT patient population in this study had overt hypothyroidism that required substitution with exogenous thyroid hormone [32,34].

## 4. Subclinical Hypothyroidism and Goiter

Beyond the most common forms of goiter mentioned above (i.e., diffuse goiter in GD and nodular goiter), various less common causes of thyroid enlargement also exist. Among them is a rare genetic disease termed familial nontoxic multinodular goiter (FNTMNG); as its name implies, it is characterized by occurrence in families (genetically transmitted in an autosomal dominant or X-linked mode), normal thyroid function, and presence of multiple nodules in the thyroid gland. One of the genes associated with this rare disease is *KEAP1*. Two loss-of-function mutations in *KEAP1* have been described, each in a respective family with FNTMNG [35,36]. The first reported family was thoroughly phenotyped, and cosegregation of the phenotype and the mutation was convincingly demonstrated [35], whereas in the latter family only the proband was genotyped [36]. Therefore, in a recent study we used Keap1^KD^ mice to verify whether decreased Keap1 levels can cause goiter. We found that Keap1^KD^ mice showed a diffuse goiter that was already detectable in early adult life and became more prominent and fully penetrant in later life [24]. Histomorphometric analysis of the thyroids showed that the goiter was characterized by markedly dilated thyroid follicles, and especially an increased size of the colloid compartment, while there were no thyroid nodules or hyperplasia. In early adult life, Keap1^KD^ mice showed decreased plasma levels of T4; these were normalized in later life, apparently by increased plasma levels of thyroid-stimulating hormone (TSH), the pituitary hormone that stimulates follicular thyroid cell growth and function. Nrf2 was activated in the thyroid of Keap1^KD^ mice, as evidenced by increased mRNA levels of its target genes *Nqo1*, *Gpx2,* and *Txnrd1*, as well as increased protein abundance of Nqo1. These findings indicate that loss of Keap1 function in mice can indeed cause goiter, though not necessarily with a nodular appearance as in the case of patients with FNTMNG. The precise mechanism accounting for the goiter phenotype and the elevated TSH levels warrants further research. Expression profiling of thyroidal genes and proteins in Keap1^KD^ mice showed changes in the abundance of the mature protein forms of several Tg-processing and Tg-degrading cathepsin enzymes; this suggests that the pathogenesis of goiter in Keap1^KD^ mice might involve increased solubilization and lysosomal degradation of Tg [24]. The phenotypes of Keap1^KD^ mice are summarized in Figure 2.

The findings from Keap1^KD^ mice suggest that chronic genetic activation of Nrf2 signaling may have negative consequences for the thyroid gland [24]. However, analysis of data from a clinical trial has shown that consumption of a broccoli sprout beverage (yielding pharmacologically active amounts of the Nrf2-activating compound sulforaphane) is safe for thyroid hormonal, and autoimmune status during a 12-week administration period [37]. Nevertheless, it appears prudent to monitor thyroid function and thyroid volume (at least by palpation) in patients treated with Nrf2-modulating compounds in clinical trials or clinical practice. In support of this concept, a recent case report described a patient with multiple sclerosis (MS) who received treatment with the approved Nrf2 activator dimethyl fumarate (DMF). The patient had concomitant HT, which showed exacerbation after 2 months on DMF, leading to treatment discontinuation. The authors postulated that HT exacerbation may be linked to the antioxidant effects of DMF, and they recommended monitoring of thyroid function tests of MS patients treated with DMF, especially when they are affected by concomitant autoimmune thyroid diseases [38].

## 5. Metabolism

Elevated TSH levels with normal T4 levels, as observed in Keap1^KD^ mice [24], are indicative of a mild form of hypothyroidism, called subclinical hypothyroidism. In this condition, increased secretion of TSH by the pituitary provides additional stimulation to the thyroid that compensates for a mildly deficient hormonogenetic capacity of the gland, thus managing to maintain T4 levels within the normal range [39]. The apparent presence of subclinical hypothyroidism in Keap1^KD^ mice [24] may have practical relevance beyond our understanding of thyroid economy, and specifically in the broader field of metabolic research. In addition to its antioxidant and cell-protective functions, the Nrf2 pathway has been implicated in metabolic regulation, either in peripheral tissues or via interactions with central signaling systems that respond to nutritional inputs [40]. In that context, Keap1^KD^ mice, as a model of constitutive Nrf2 pathway activation, have been shown to be protected against metabolic disease (obesity, insulin resistance, type 1 and type 2 diabetes). This effect has been attributed not only to the classic protective effects of Nrf2 against oxidative stress damage in the hypothalamus [41] and in β cells [42,43], but also to novel roles of Nrf2 in the repression of gluconeogenic [44,45] and lipogenic [37] pathways. Of note, none of these metabolic studies assessed the thyroidal status of the respective animals. As it is practically impossible to precisely predict if and how an experimental diet (e.g., high-fat, high-sucrose, etc.) or a disease-related genetic background (e.g., lipodystrophy, *db*/*db*, etc.) may affect the timing and severity of thyroid dysfunction specifically in Keap1^KD^ mice, we suggest that metabolic studies using these animals should routinely evaluate thyroidal status in all experimental groups. Conversely, only two studies have evaluated energy expenditure in Keap1^KD^ mice, one using 4-month-old male mice on a high-fat diet for 2 months [45], and the other using 3-month-old male mice in a lipodystrophy background [37]. In these studies, hypomorphism for Keap1 did not significantly alter energy expenditure in the context of the respective disease model, but neither study directly compared Keap1^KD^ mice to WT controls without other genetic or pharmacological interventions [37,45]. Thus, the possible metabolic correlates of subclinical hypothyroidism in Keap1^KD^ mice warrant further investigation, and such studies might help to address the existing controversy regarding the effects of moderate, subclinical thyroid dysfunction on energy expenditure [46].

Lastly, an interesting observation came from a study in mice that found that administration of a high-fat diet decreased the nuclear levels and increased the cytoplasmic levels of Nrf2 in thyroid follicular cells, indicating a suppression of Nrf2 activity. This effect could be abrogated by various interventions, including a low-fat diet, caloric restriction, exercise, or treatment with the Nrf2 activator quercetin. The expression levels of microRNA *miR-200a* in the thyroid of high-fat diet-treated mice were increased [47], which might have a causal effect on the activity of Nrf2, given that *miR-200a* has been previously shown to target *Keap1* [48].

## 6. Hyperthyroidism/Thyrotoxicosis and Graves’ Orbitopathy

In contrast to hypothyroidism, hyperthyroidism and thyrotoxicosis are characterized by excessive production and/or liberation of thyroid hormones. Like hypothyroidism, hyperthyroidism is more common in women; the most common cause in younger patients is autoimmune thyroid disease (specifically GD), whereas in older patients it is hyperfunctioning thyroid nodules, either single (toxic adenoma) or multiple (toxic multinodular goiter) [19]. Regardless of the cause, the high concentrations of circulating thyroid hormones result in overstimulation of peripheral tissues, inducing an overall increase in metabolism and an increased demand for energy and oxygen. Therefore, it is no surprise that hyperthyroidism/thyrotoxicosis has also been associated with OS and with activation of Nrf2 signaling in the target tissues of thyroid hormones. Specifically, it has been known for several years that thyroid hormone administration induces activation of Nrf2 in the rat liver, and that this activation is suppressed by pretreatment with the ROS scavenger N-acetyl cysteine [49]. In the same line, it was recently shown that in the lymphoid tissues of mice treated with high doses of exogenous T4, thyrotoxicosis induces an increase in ROS levels, activates Nrf2, and upregulates the transcription of genes encoding antioxidant enzymes [50]. Activation of Nrf2 by thyroid hormone was further confirmed in other recent studies focusing on the heart [51] and the liver [52].

Some studies have found that natural antioxidants, such as curcumin and vitamin E, can decrease OS in the peripheral tissues of animals with T4-induced experimental thyrotoxicosis [53], and can prevent the marked activation of the antioxidant response [54]. A recent study assessing these effects in the rat heart employed in vitro modelling to suggest that they may involve modulation of Keap1/Nrf2 signaling by curcumin and vitamin E [55]. In another recent study, quercetin, a flavonoid with antioxidant properties, was found to protect rats from thyrotoxicosis-induced liver damage [56]. As quercetin is a known Nrf2 activator, the study also employed Nrf2 KO rats and found that the protective effects of quercetin against thyrotoxicosis-induced liver damage were diminished in these animals, which suggests that they may be mediated by Nrf2 [56].

In GD, the autoimmune reaction does not target only the thyroid, causing hyperthyroidism, but occasionally also the eye and its surrounding tissues (muscles and fat), causing Graves’ orbitopathy (GO), a sight-threatening ocular disease [57]. While the pathogenesis of GO is not well understood, it is known to involve strong components of OS, inflammation, and periorbital fat accumulation, as well as to have a strong clinical correlation with smoking [58]. Treatment of mild forms mainly includes smoking cessation and oral administration of the antioxidant selenium, whereas treatment of moderate and severe forms is problematic and is based on corticosteroids and targeted immunomodulatory agents [58]. Thus, GO, albeit rare, continues to represent an important unmet medical need. A recent study using a mouse model of GO found that polydatin, a natural antioxidant, can activate Nrf2, upregulate antioxidant genes, reduce the production of ROS, and inhibit adipose tissue expansion and lipid droplet accumulation in vivo [59]. In addition, in orbital fibroblasts cultured in vitro, silencing Nrf2 reduced the protective effects of polydatin against H_2_O_2_-induced OS [59]. This is the first study to address the involvement of Nrf2 and its modulators in GO, and it is hoped that further research in this field can yield new approaches to combat this potentially sight-threatening disease.

We have recently reviewed the effects that various natural compounds, including Nrf2 activators, can have on the thyroid gland to favor or suppress thyroid hormone production, with potential utility against hypothyroidism or hyperthyroidism, respectively [60]. The aforementioned recent studies further show that such compounds may also have utility in counteracting the manifestations of thyroid dysfunction in peripheral tissues.

## 7. Thyroid Carcinomas

Most thyroid carcinomas are generally associated with a better prognosis compared to other types of cancer, yet the disease and its treatment are often associated with substantial morbidity as well as a negative impact on the patient’s quality of life. The favorable prognosis applies especially to differentiated thyroid carcinoma (DTC), which is the most frequent type, and originates from thyroid follicular cells, whereas other types like poorly differentiated thyroid carcinoma (PDTC, likely representing an evolution of DTC) and medullary thyroid carcinoma (MTC, originating from calcitonin-secreting parafollicular cells that reside between the thyroid follicles) have a poorer prognosis, and anaplastic thyroid carcinoma (ATC) is almost always lethal (recently reviewed in [61]). The most common type of thyroid cancer is papillary thyroid carcinoma (PTC), which is a form of DTC [23]. Activation of Nrf2 is generally known to play a dual role in cancer, on the one hand protecting normal cells against oxidative damage and thereby preventing carcinogenesis, while on the other hand conferring a survival advantage to established cancer cells [62]. In our previous review on the roles of Nrf2 in the thyroid, we have thoroughly reviewed the studies examining its implication in PTC and other types of thyroid cancer [5]. In summary, we had initially observed that Nrf2 is commonly activated in PTC, where it regulates the antioxidant defense and promotes the survival of cancer cells [63]. This observation was subsequently confirmed by other groups [64], and the underlying molecular mechanisms began to be unraveled; these include somatic mutations in *KEAP1* [65], epigenetic hypermethylation of *KEAP1* or other genes in the Nrf2 pathway [64], etc. In the present work, we review a few recent studies that have shed further light into the involvement of Nrf2 in thyroid cancer.

Transcriptomic profiling of PTC samples compared to adjacent normal thyroid tissues identified the Nrf2-mediated oxidative stress response as one of six commonly upregulated oncogenic pathways [66]. Somatic mutations in *KEAP1*, *NFE2L2*, or other Nrf2 pathway components were not reported in this study, which is not surprising, knowing that the frequency of such mutations in thyroid cancer is lower than in other cancer types with activated Nrf2 signaling [64,65], and given the small sample size of the specific study [66]. Another study found higher *Nrf2* mRNA levels and Nrf2 protein abundance in PTC samples compared to normal tissues [67]; of note, Nrf2 is known to induce the transcription of its own gene (at least in the mouse), via an ARE element in the gene promoter [68]. Further, the same study showed that inhibition of microRNA *miR-17-5p* in a PTC cell line decreased *Nrf2* mRNA expression levels [67]. Another study found increased expression of the long non-coding RNA lung cancer associated transcript 1 (*LUCAT1*) in PTC, as well as a positive correlation between *LUCAT1* expression levels and unfavorable clinicopathological parameters [69]. In two PTC cell lines, knockdown of *LUCAT1* decreased *Nrf2* mRNA expression levels and Nrf2 protein abundance, while at the same time it reduced cell proliferation and invasion, and induced cell-cycle arrest and apoptosis [69]. Lastly, another study showed that Pinelliae rhizome (PR), a component of a traditional medicine decoction, decreased the protein abundance of Nrf2 in the nucleus of PTC cell lines in a dose-dependent manner, and decreased their viability [70]. Knockdown of Nrf2 via small interfering RNA (siRNA) in the PTC cell lines decreased their viability and had an additive or synergistic effect with PR. In mice orthotopically transplanted with a PTC cell line, the combination of Nrf2 knockdown in the PTC cells before transplantation and PR treatment of the animals significantly decreased the tumor burden and extended the survival of the animals. In contrast, when the PTC cells were transplanted into the thyroids of Nrf2 KO mice, the tumor burden was increased, survival was shortened, and the protective effect of PR treatment was lost [70]. Taken together, the findings of the aforementioned recent studies further support the original concept that the Nrf2 pathway is activated in PTC and has a protective effect in thyroid carcinoma cells [63].

Preclinical data have suggested that dipeptidyl peptidase-4 (DPP-4) inhibitors, a glucose-lowering treatment for diabetes, may promote metastatic progression of preexisting cancer via activation of Nrf2 in the cancer cells [71]. However, a subsequent population-based clinical study from Germany did not find an association between DPP-4 inhibitor treatment and cancer metastasis [72]. Recently, another clinical study from Korea tested for associations between glucose-lowering drugs and cancer metastasis in several cancer types; no associations were found, except for a weakly significant association between DPP-4 treatment and thyroid cancer metastasis (hazard ratio 3.89, confidence interval 1.04–9.64) [73]. The authors acknowledged that this finding should be taken with caution, because the number of patients with thyroid cancer in the study was small, and the frequency of thyroid cancer metastasis was low in all patient groups [73]; it might thus represent a spurious association, and further studies are required to confirm or refute it.

## 8. Concluding Remarks

The Keap1/Nrf2 system is known to play important roles in redox balance and metabolic homeostasis, with documented beneficial effects in models of inflammation, autoimmunity, metabolic, and neurodegenerative diseases, and many other chronic conditions [14]. The present focused review of the thyroid field suggests that the Keap1/Nrf2 system might also have utility in the search for biomarkers and/or drug targets for thyroid pathologies. In view of the current state of knowledge as outlined above, in our opinion, some of the main areas for future research around Nrf2 and thyroid are the following: (i) further elucidation of the mechanisms by which Nrf2 signaling impacts the thyroid hormone synthesis and secretion apparatus; (ii) documentation of Nrf2 activity status in models of hyperthyroidism/thyrotoxicosis, hypothyroidism, and goiter; (iii) in vivo characterization of the effects of Nrf2-activating antioxidant substances on the thyroid; (iv) investigation of the potential utility of Nrf2 modulation in the context of thyroid autoimmunity; and (v) elucidation of the potential role of altered thyroid function in the various models studying the implication of Nrf2 in metabolic disease. Our ongoing research program aims to address specifically the aforementioned knowledge gaps, with the ultimate goal of devising new strategies to improve the prevention and treatment of thyroid diseases.

## Abbreviations

AGEs	advanced glycation end products
ARE	antioxidant response element
CRISPR/Cas9	clustered regularly interspaced short palindromic repeats/CRISPR-associated protein 9
CUL3	cullin 3
DPP-4	dipeptidyl peptidase-4
DTC	differentiated thyroid carcinoma
FNTMNG	familial nontoxic multinodular goiter
GD	Graves’ disease
GO	Graves’ orbitopathy
Gpx2	glutathione peroxidase 2
H_2_O_2_	hydrogen peroxide
HT	Hashimoto’s thyroiditis
Keap1	Kelch-like erythroid cell-derived protein with cap’n’collar homology-associated protein 1
Keap1^KD^	Keap1 knockdown
LUCAT1	lung cancer associated transcript 1
Maf	musculoaponeurotic fibrosarcoma
MS	multiple sclerosis
*NFE2L2*	nuclear factor erythroid 2-like 2
NIS	sodium-iodide symporter
Nqo1	NAD(P)H quinone dehydrogenase 1
Nrf2	nuclear factor erythroid 2-related factor 2
Nrf2 KO	Nrf2 knockout
OS	oxidative stress
ROS	reactive oxygen species
PTC	papillary thyroid carcinoma
RAGE	receptor for advanced glycation end products
*SELENOS*	selenoprotein S
Sels	selenoprotein S
siRNA	small interfering RNA
SNP	single-nucleotide polymorphism
T3	triiodothyronine
T4	thyroxine
Tg	thyroglobulin
TSH	thyroid-stimulating hormone
Txnrd1	thioredoxin reductase 1
WT	wild type
